# Host cell protein platform assay development for therapeutic mAb bioprocessing using mammalian cells

**DOI:** 10.1186/1753-6561-9-S9-P21

**Published:** 2015-12-14

**Authors:** Nadine Kochanowski, Gaetan Siriez, Larissa Mukankurayija, Aurélie Delangle, Alex Murray-Smith, Kevin Dromer, David Mainwaring, Clemens Stilling, Nadja Prang, Laetitia Malphettes, Annick Gervais

**Affiliations:** 1 Upstream Process Sciences Group, Biotech Sciences, UCB Pharma S.A., Braine L'Alleud, 1420, Belgium; 2 Analytical Sciences for Biologics, Biotech Sciences, UCB Pharma S.A., Braine L'Alleud, 1420, Belgium; 3 Downstream Process Sciences, Biotech Sciences, UCB Pharma S.A, Slough, SL13WE, UK; 4 Protein Expression and Purification, UCB Celltech, Slough, UK; 5 TECObiosciences, Rheinbach, 53359, Germany

## Background

Recombinant therapeutic proteins are usually produced by cell culture technology using genetically modified host cell lines. During the manufacturing process, a mixture of the protein of interest and host cell derived impurities, including host cell proteins (HCPs) and other process related impurities are produced. Those process related impurities will be cleared or minimized though the process by optimization of process purification. Residual HCPs in the final drug substance may affect the quality, safety and efficacy and may result in clinical adverse effects. HCPs are typically quantified using immunoassays such as enzyme-linked immunosorbent assay (ELISA). The development and the validation of those assays are really challenging mainly due to the wide variety of possible HCPs in products. Although generic ELISA kits are commercially available to quantify HCPs from different recombinant systems, a process specific assay is required before drug registration and commercialization for biologics. The reagents, polyclonal antibodies and HCP standards, need to be carefully prepared and characterized to ensure a correct quantitation of the HCPs in the final product. Here we describe the first step of the development of a production platform-specific HCP-ELISA assay for UCB's biopharmaceuticals produced in a Chinese Hamster Ovary (CHO) cell line, i.e. the production of mock material containing the HCPs using a null cell line.

## Materials and methods

2L and 80L stirred tank bioreactors (Sartorius) were run for 14 days in a fed-batch mode in a chemically defined medium. Feed was added daily from day 3 onwards. If required, antifoam was added to the bioreactor by manual injections. Dissolved Oxygen (DO), pH, and temperature were controlled at set points. DO was controlled using a multi-stage aeration cascade via a ring sparger. Viable cell concentration and cell viability were measured using a ViCell cell counter (Beckman Coulter). The osmolality was measured using an osmometer (Advanced Instruments).The off-line pH was measured using a BioProfilepHOx (Nova Biomedical). The glucose, lactate, glutamine and ammonia concentrations were measured with a BioProfile Analyzer 400 (Nova Biomedical). On the day of harvest, the clarification was performed by centrifugation, depth filtration and sterile filtration. Part of the material was further clarified by tangential flow filtration (TFF) on the Uniflux 10 system (GE Life Sciences) using low molecular weight cut-off membranes and concentrated before being diafiltered into an appropriate buffer.

The antigens from the mock run were assessed for their total HCP content by 2D DIGE and analyzed by the DeCyderTM 2D 7.2 software.

## Results

**Figure 1:**
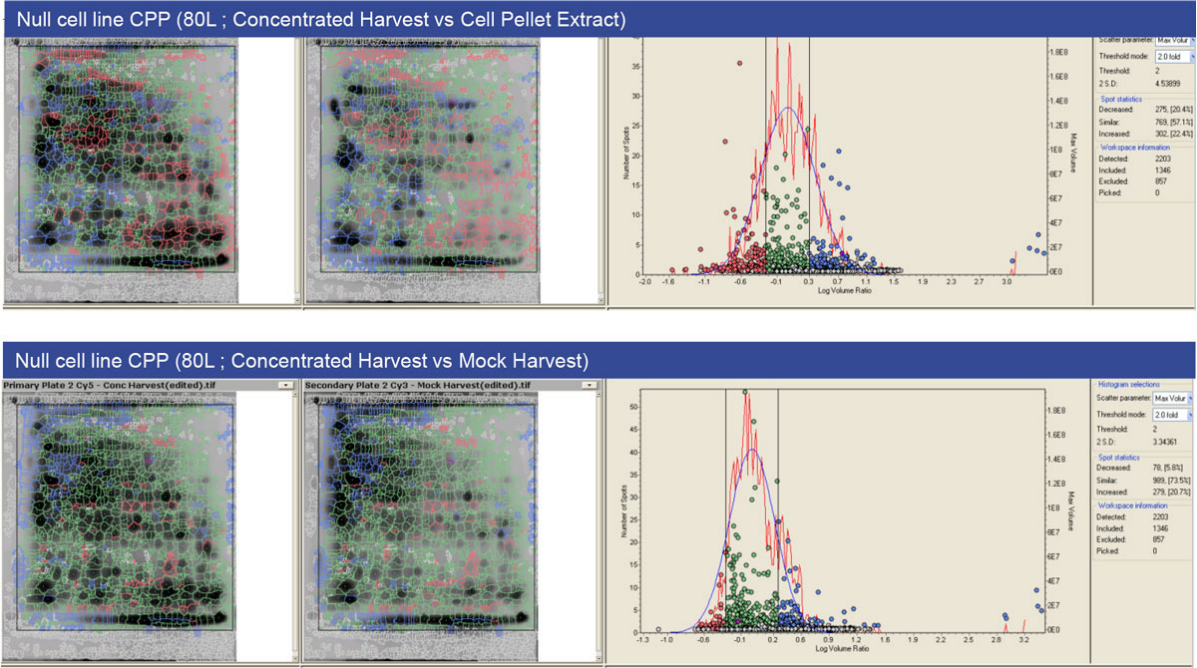
**In-gel comparison of concentrated Harvest material *versus *cell pellet material and mock harvest material**.

Different feeding strategies have been assessed at 2L scale to select the best condition enabling obtaining comparable cell growth and metabolite profiles between the null cells and three cell lines derived from our recombinant platform and expressing different mAbs (Cell lines mAb1, mAb2 and mAb3). The three feeding strategies assessed were the center point process (CPP), CPP+20% and CPP-20%. Viable cell density, cell viability, off-line pH, glucose, lactate glutamine, ammonia and osmolality levels have been monitored throughout the cultures and the HCP profiles of the cell pellet and supernatant material were assessed by 2-D Fluorescence Differential Gel Electrophoresis (2D-DIGE) analysis. The gels of each of the samples were analyzed to assess the spot coverage percentage and to obtain a qualitative HCP profile of each of supernatant and cell pellet. These profiles were reproducible between gels which meant both in-gel and inter-gel analysis could be performed. Based on the spot coverage (Table 1) and the intensity, the HCP profiles were comparable with slight differences between the feeding strategies. Based on the process performance output parameters and HCP profiles, the Center Point Process (CPP) feeding strategy was selected to be applied on the mock production in 80L stirred tank bioreactors. The null cell line output parameter profiles were comparable between the two scales, 2L and 80L bioreactors. Moreover, the cell growth profiles of the mock run were comparable to the antibody producing cell lines. After mock materiel production at 80L scale, the mock harvest, the concentrated harvest and the cell pellet were compared using 2D-DIGE to evaluate the common spots between the different antigens. The comparison has been made against the concentrated harvest material as this is most representative of the total HCP profile (Figure 1). Around 57% of the spots found in the cell pellet extract were similar to those found in the concentrated harvest whereas around 72% of the mock harvest spots were similar to concentrated harvest spots.

**Table 1:** Comparison of spot coverage within the same gel of the supernatant and the cell pellet samples, for both feeding strategies CPP and CPP-20%

	Null cell line CPP (2L scale)	Null cell line CPP-20% (2L scale)
**% of spot coverage in the supernatant**	**71**	**72**
**% of spot coverage in the supernatant**	**65**	**64**

## Conclusions

A crucial step of the development of a platform HCP-ELISA assay is to set-up the mock cell run conditions to obtain comparable cell growth, hence comparable HCP profiles the null cell and production cell lines. Here, we optimized the mock upstream conditions at 2L scale and scaled up at 80L scale. We demonstrated that the mock cell line growth and HCP profiles are comparable at both scales, 2L and 80L respectively. In addition, we have shown that the HCP profiles were also comparable among three different production cell lines. Consequently, the generated mock material was used for the generation of the anti-HCP serum that will allow the development of a UCB platform assay for determination of HCP content. For each new biological produced with UCB's recombinant expression platform, the 80L mock growth and HCP profiles will be compared with the 80L production profile to ensure that the platform assay is suitable for the new molecule.

